# Interannual variability in net ecosystem carbon production in a rain-fed maize ecosystem and its climatic and biotic controls during 2005–2018

**DOI:** 10.1371/journal.pone.0237684

**Published:** 2021-05-10

**Authors:** Hui Zhang, Tianhong Zhao, Sidan Lyu, Hang Wu, Yang Yang, Xuefa Wen

**Affiliations:** 1 College of Agronomy, Shenyang Agricultural University, Shenyang, China; 2 Jinzhou Ecology and Agriculture Meteorological Center, Liaoning Meteorological Bureau, Jinzhou, China; 3 Key Laboratory of Ecosystem Network Observation and Modeling, Institute of Geographic Sciences and Natural Resources Research, Chinese Academy of Sciences, Beijing, China; Tennessee State University, UNITED STATES

## Abstract

Interannual variability (IAV) in net ecosystem carbon production (NEP) plays an important role in the processes of the carbon cycle, but the long-term trends in NEP and the climatic and biotic control of IAV in NEP still remain unclear in agroecosystems. We investigated interannual variability in NEP, expressed as annual values and anomalies, and its climatic and biotic controls using an eddy-covariance dataset for 2005–2018 for rain-fed spring maize in northeastern China. Average annual NEP was 270±31 g C m^−2^yr ^−1^, with no significant changes over time. The effects on interannual variability in NEP of gross ecosystem productivity (GEP) that was mainly controlled by soil water content (SWC) and leaf area index (LAI), were more than those of respiration (RE) that was controlled by temperature and LAI. Further, maximum daily NEP (NEP_max_) that was dominated by summer vapor pressure deficit explained the largest fraction of annual anomalies in NEP, followed by carbon dioxide uptake period (CUP) that was defined by the beginning date (BDOY) and the end date (EDOY) of CUP. The variability in BDOY was mainly determined by spring precipitation and the effective accumulated temperature, and the variability in EDOY was determined by autumn precipitation, SWC and LAI. NEP may decrease with declining precipitation in the future due to decreasing GEP, NEP_max_, or CUP, and irrigation and residues cover may be useful in efforts to maintain current NEP levels. Our results indicate that interannual variability in NEP in agroecosystems may be more sensitive to changes in water conditions (such as precipitation, SWC and VPD) induced by climate changes, while temperature may be an important indirect factor when VPD is dominated.

## Introduction

Cultivated land occupies approximately 40% of the terrestrial surface, and 30% of that is used for agriculture [1]. The carbon status of agricultural ecosystems may fluctuate between a net source and sink, or it may remain neutral [2]. In the Northern Hemisphere, croplands contribute 21% to total long-term carbon uptake, and 39% to the interannual variation (IAV) in net ecosystem carbon production (NEP) of terrestrial ecosystems [3, 4]. The interannual variability in NEP, expressed as annual values and anomalies, reveals the variation in the length and intensity of the active periods of photosynthesis and respiration, which determine carbon dioxide sink or source in agroecosystem [5, 6]. Changes in vegetation phenology and physiological processes, driven by the variability in climatic and biotic factors, lead to interannual variability in NEP [7]. However, the key factors and processes determining the interannual variability in NEP are far from clear, because many physical and biological forcing are changing simultaneously and they can affect the bidirectional fluxes, photosynthesis and respiration, in distinct and offsetting way in different ecosystems [8].

Trends in NEP may contain large uncertainty due to the minor difference between the interannual variability in NEP and the detection limit of NEP trends, and the short length of time series [8, 9]. A trend analysis used in conjunction with a literature review of interannual variability in carbon fluxes showed that the detectable trends thresholds reduced markedly as the time series extends from 3 to 10, 12 and 13 years if the measurement uncertainties were 10, 30 and 60 g C m^−2^ year^−1^, respectively, and had little reduction when the time series were longer than the decade [8]. In addition, the detectable trends thresholds increased with the increasing of measurement uncertainties, and the net ecosystem carbon exchange might exceed 3, 6 and 10 g C m^−2^ year^−2^ to be significantly different than 0 when the time series is at least a decade in duration and the measurement uncertainty is ± 10, 30 and 60 g C m^−2^ year^−1^, respectively [8]. After excluding highly disturbed plantation sites, 26 studies based on a time series of a decade or longer indicated a trend in net ecosystem exchange (NEE) of −8.9 ± 7.5 g C m^−2^ year^−2^, which was not significantly different from the detection limit of the methods [9]. In addition, long-term trends in carbon fluxes can reverse when time series are extended. Based on 13 years (1992–2004) of carbon flux measurements at the Harvard Forest, MA, USA, long-term increasing trends in NEE were interrupted in 1998 by a sharp decline, with a recovery over the subsequent 3 years [10].

The climatic and biotic control of interannual variability in NEP is complex and unclear. Climatic and biotic factors change simultaneously, and can affect bidirectional fluxes (photosynthesis and respiration) in distinct and offsetting ways in different climates and ecosystems [11]. To increase the understanding of the climatic and biotic control on the interannual variability in NEP, NEP can be partitioned into gross ecosystem productivity (GEP) and ecosystem respiration (RE) [11–15], and further be divided into carbon dioxide flux uptake (NEP>0) or release (NEP<0) peak (plant physiology indicator) and corresponding periods (phenological indicator) [3, 16–18]. Compared to RE, anomalies in NEP may be more sensitivite to the climatic and biotic factors of driving GEP, as indicated by a dataset of 544 site-years from 59 sites around the world [4]. The influences of climatic and biotic factors on GEP and RE were different for different ecosystems, regions, and climate conditions at long timescales [19, 20]. Precipitation increased GEP and aboveground biomass from 2006 to 2015 in three different agroecosystems with maize, soybeans, and tallgrass prairie in the USA; this increased residues that were the aboveground biomass leaving on the bare soil surface after crop harvest [21]. In addition, the effects of drought stress on GEP were more than those on RE in grasslands of the Loess Plateau, and the sensitivity of RE to soil temperature might be decreased with the reduction of soil moisture while RE was mainly affected by soil temperature [22].

**Fig 1:**
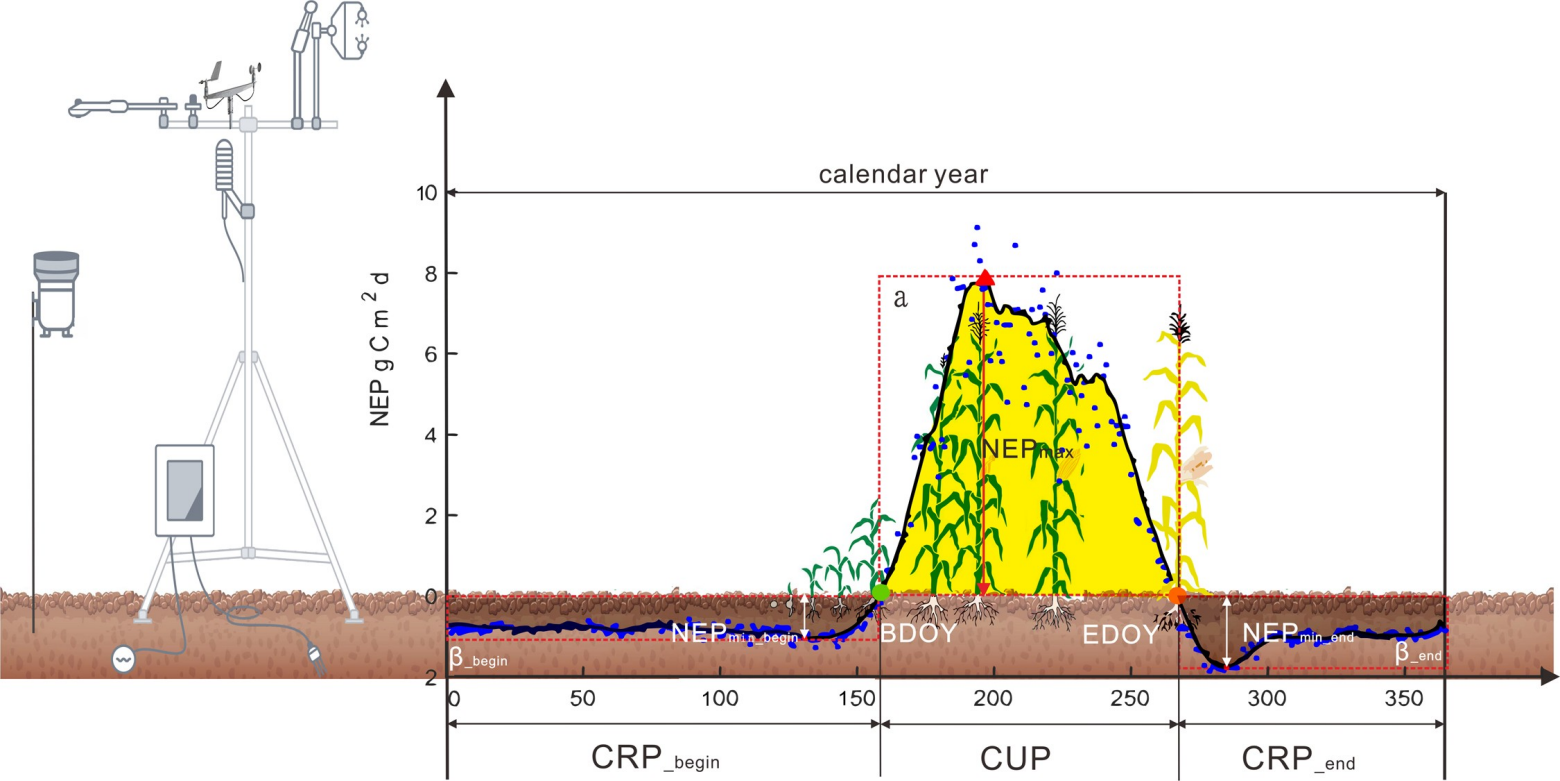
Schematic diagram of variables determining changes in annual net ecosystem CO_2_ exchange, including the ratio of actual carbon dioxide uptake to hypothetical maximum carbon dioxide uptake (α), maximum daily NEP (NEP_max_), carbon dioxide uptake period (CUP), and the ratio of actual carbon release to hypothetical maximum carbon release (β), minimum daily NEP (NEP_min_) and carbon release period (CRP) at the beginning and at the end of the year. Blue points indicate average daily NEP from 2005 to 2018 in this study; the black line shows 10-day averages.

Net carbon dioxide uptake and release period (CUP and CRP), and the maximum and minimum daily NEP (NEP_max_ and NEP_min_) result from different mechanisms through which climatic and biotic factors affect net carbon uptake physiology and phenology, and then regulate the interannual variability in NEP, and NEP = f (CUP, NEP_max_, CRP, NEP_min_) (Fig 1) [16–18]. Because climatic and biotic factors have compensatory effects on the NEP_max_/ NEP_min_ and CUP/CRP, negligible impacts on annual NEP and conflicting results have been described in previous studies [23]. The carbon dioxide release period, especially in agroecosystems, is not always continuous within a calendar year [24], and this was often ignored in previous studies [21, 25–27]. According to an atmospheric inversion dataset and a global analysis of NEP, the large interannual variability in NEP was mainly attributed to both an extended CUP and an increasing amplitude of NEP_max_ [18]. Based on long-term NEP calculated from 66 eddy covariance sites and global products derived from FLUXNET data, global scale NEP was predominately determined by NEP_max_ [18]. 31% of the interannual variability in NEP in water-limited ecosystems was determined by interannual variability in CUP, and 60% of that in temperature and radiation-limited ecosystems was determined by the interannual variability in NEP_max_ [18]. NEP during the carbon release period explained 10% of the variability in annual NEP, using long term observed NEP from 66 eddy covariance sites and global products derived from FLUXNET observations. Meanwhile for the agroecosystems, the explanation of NEP during the carbon release period for the variability in annual NEP incresed to 24%, which controlled by air temperature and aboveground residues after crop harvest [3, 28].

Rain-fed spring maize is the dominant crop in northeastern China, accounting for 57.2% of the planted area, 64.3% of the crop yield, and 33.8% of the total maize yield in China [29, 30]. The NEP for agroecosystem in the Northeast China fluctuated between 0.007 and 0.0031 Pg C a^-1^ during the past decade, with an average value of 0.026 Pg C /a, which accounted for 18.3% of NEP for agroecosystem in China [29]. Besides NEP, the carbon balance contains the non-respiratory carbon losses (harvest) as well as non-photosynthetic carbon gains (organic fertilisation), resulting in the net biome productivity (NBP) [28]. The previous study showed that NBP positively correlated with NEP (R^2^ = 0.35, P<0.001) according to a dataset of 103 site-years from 14 crop sites around the world [2, 13, 21, 22, 25, 28]. Therefore, the agroecosystem in the area has a potentially large capacity for carbon sequestration or release depending on climatic and biotic factors. A prolonged drought affected 52.3–62.0% of the area in northeastern China in the past 15 years [31], and there is an increasing trend in temperature of 0.035°C/a and a decreasing trend in precipitation of −13.3 mm/10a [29]. An increased understanding the relationships between interannual variability in NEP and climatic and biotic controls will aid in efforts to improve the carbon sequestration potential. We conducted the continuous measurements of carbon dioxide fluxes using eddy covariance in rain-fed spring maize in northeastern China since June 2004. The interannual variability in NEP, expressed as annual values and anomalies, was investigated during 2005–2018. NEP was partitioned into GEP and RE, and the NEP_max_\NEP_min_ and CUP\CRP. Our objectives were to 1) identify the long-term trend and interannual variability in NEP, and 2) to illustrate the climatic and biotic controls on interannual variability in NEP partitioned into GEP and RE, and NEP_max_\NEP_min_ and CUP\CRP.

## Materials and methods

### Site characteristics and agricultural management

Eddy fluxes of carbon dioxide and water vapor were measured in a typical rain-fed maize agroecosystem at the Jinzhou site (41°08’ N, 121°12’E, and 23.3 m above sea level) in northeastern China as a part of the China FLUX network. This flux site is located in a representative temperate monsoon climate area with a mean annual temperature of 9.4°C and annual precipitation of 565.9 mm, based on long-term records (1981–2010) from an adjacent weather station [32]. The average rate of air temperature increase is 0.035°C/a, and average precipitation decrease is 13.3 mm/10a, based on the 1961–2010 ground observations at 91 meteorological stations [29]. There are two prevailing wind directions, including north-northeast in winter and south-southeast in summer.

Rain-fed maize is generally sown every year during mid-April to mid-May without rotation, and harvested during mid-September to early October (detail in S1 Table), depending on air temperature and natural soil moisture conditions. During the study period, maize was cultivated at an average density of 45,800 plants per ha. The maximum leaf area index (LAI) was 4.4 during the tasseling period and the maximum canopy height was 2.8 m during the filling period, respectively. During the off-season from September/October to April/May, maize harvest residues, including aboveground stems and leaves, were left directly on the bare soil surface, while grain was removed from the agroecosystem. Bulk density of the loamy soil was 1.6 g cm^−3^, pH was 6.4, and soil organic carbon was 23.6 g kg^−1^ [32]. Chemical fertilizer (NH_4_HCO_3_) was applied only during planting at concentrations of 1000 kg ha^−1^ [33].

### Eddy covariance and auxiliary measurements

#### Eddy covariance measurements

The eddy covariance (EC) system was equipped with an open path infrared gas analyzer (LI-7500, Licor Biosciences Inc.) and a 3-D sonic anemometer (CSAT, Campbell Scientific Inc.), which were installed at a fixed height of 4 m. Sampling frequency was 10 Hz and all signals were recorded on data loggers (CR5000, Campbell Scientific Inc.) starting in June 2004. The gas analyzer was periodically calibrated to avoid instrument drift. The flux tower was located approximately in the center of the maize field with at least a 380 m radius. The representative area of the flux footprint climatology (S1 Fig) was 0.02–0.34 km^**−2**^ in the growing season, and 0.39–0.42 km^**−2**^ in the non-growing season, calculated by a footprint model proposed by Kormann and Meixner using data from 2005–2018 [34, 35].

#### Auxiliary meteorological, and soil and plant measurements

Auxiliary radiation measurements were conducted using a 4-component net radiometer (CNR-1, Kipp & Zonen), and a quantum sensor of photosynthetically active radiation (PAR) (Li190SB, LI-COR Inc.), positioned over the canopy at heights of 5 and 4 m, respectively. Precipitation (PPT) was monitored with a tipping bucket (52202, RM Young Inc). Air temperature (Ta) and relative humidity were measured with an HMP45C (Vaisala, Campbell Scientific Inc.) at heights of 4 and 6 m. Wind speed and direction were measured with a wind sensor (034B, MetOne Inc.) at 6 m above ground. Soil volumetric water content (SWC) at 10 cm depth was measured with Eazy-AG50 (Sentek Inc.), and soil temperature (Ts) at 5, 10, 15, 20, 40, and 80 cm depths were obtained with HFT01SC (Campbell Scientific Inc.). All of the meteorological and soil variables were recorded using a datalogger (CR23X, Campbell Scientific Inc.).

Leaf area index (LAI) was determined by randomly sampling 5 maize plants, and manually measuring the lengths and widths of every leaf at three-leaf, seven-leaf, jointing, tasseling, and filling stages, respectively. The leaf area of each plant was obtained by adding leaf lengths multiplied by widths and a maize coefficient of 0.7 [36, 37], and then LAI at every growth stage was calculated by multiplying the average leaf area of samples with plant density. The Normalized Difference Vegetation Index (NDVI, 16 days, 250 m, MOD13Q1) was downloaded from the National Aeronautics and Space Administration (NASA) website (https://modis.gsfc.nasa.gov/data/), and then the average NDVI during each maize growth stage was obtained according to the NDVI of 16 days. The 16-day LAI was calculated based on the correlation between LAI and NDVI for each maize growth stage (S2 Table), and then daily LAI was obtained by linearly interpolating the 16-day LAI.

In addition, the residues (Resi) indicated as the carbon content of leaves and stems left on the bare soil surface after harvest during the off-season, and the roots were ignored. Forty maize plants after maturity were randomly sampled and divided into grains, stems, and leaves after 30 days of air-drying. Corresponding weights per unit area were obtained by multiplying by plant density. Carbon content of leaves and stems was determined by multiplying dry weights by 0.4 [38].

### Flux data processing and calculations

#### Data processing and quality control

Net exchange production (NEP, g C m^**−2**^ s^**−1**^) was measured using the EC technique, and its calculation equation is as:

$$NEP=-(\bar{\rho d}\bar{w^{'}s_{c}{}^{'}}+\int_{0}^{z}\bar{\rho d}\frac{\partial \bar{s_{c}}}{\partial t}dz+\int_{0}^{z}\bar{\rho d}(\bar{u}\frac{\partial \bar{s_{c}}}{\partial x}+\bar{v}\frac{\partial \bar{s_{c}}}{\partial y})dz+\int_{0}^{z}\bar{\rho d}\bar{w}\frac{\partial \bar{s_{c}}}{\partial z}dz+\int_{0}^{z}\bar{\rho d}(\frac{\partial \bar{u^{'}s_{c}{}^{'}}}{\partial x}+\frac{\partial \bar{v^{'}s_{c}{}^{'}}}{\partial y})dz)$$ (1)

where the terms on the right side of the equation are eddy flux (Term I), storage (Term II), horizontal advection (Term III), vertical advection (Term IV), and horizontal flux divergence (Term V) [39], respectively. Terms III—V were ignored because the site had negligible slope and was assumed to be homogeneous [40]. Because measurement height is low in this agroecosystem, Term II was small and therefore neglected in this study.

For the 10 Hz raw data, statistical tests were used to remove spikes [41]. Two-dimension coordinate rotation [42] was conducted to achieve the consistency of coordinates between the instrument and the flow. A Webb, Pearman & Leuning correction was applied to remove the effect of fluctuations in air density on fluxes [43]. Low and high frequency spectral corrections were also calculated [44]. The 0-1-2 flag system developed by Mauder and Foken [45] based on steady state and turbulent conditions were used to classify flux quality. Data classified with flag 2 were removed. When friction velocity (u*) was < the u*-threshold, flux data were also rejected to avoid possible underestimation of flux during stable conditions at night. The Reichstein’s method was used for determining the u*-threshold, and it firstly divided the night-time fluxes into four periods with 3 months each periods, and secondly divided air temperature into the six classes according to quantiles and defined the u*-threshold in each period by the median of the thresholds in the six classes, and lastly determined the u*-threshold for the whole data set by the highest threshold during the four periods [46].These processes were operated in EddyPro software (version 6.2.2, LI-COR, Lincoln, NE). The number of effective data for years 2005–2018 after quality control is shown in S3 Table.

#### Gap filling and flux partitioning

To acquire a continuous dataset and to estimate annual fluxes, marginal distribution sampling was used to fill data gaps [46]. The NEP was partitioned into GEP and ecosystem respiration (RE). GEP was calculated as the sum of NEP and RE.

Nighttime NEE was described as RE, because photosynthesis does not occur at night. The RE was calculated using the Lloyd and Taylor equation [47]:

$$RE=RE_{ref}\exp(E_{0}(\frac{1}{(T_{ref}+46.02}-\frac{1}{\left(T_{a}+46.02\right)}))$$ (2)

where T_*ref*_ is the reference temperature (10°C), E_0_ is the temperature sensitivity coefficient (°C) and RE_*ref*_ (g C m^−2^ s^−1^) is the reference respiration at T_*ref*_. RE_ref_ and E_0_ were estimated using regression analysis every 15 days [46], and RE of daytime was calculated. The above processes were conducted using Tovi software (version 2.7.2, LI-COR, Lincoln, NE).

### Uncertainty analysis of carbon fluxes

The uncertainty of 30-min carbon dioxide fluxes consists of (i) random errors associated with the measurement equipment, flux footprint heterogeneity, and variation in turbulent transport; (ii) fully systematic bias caused by longer gap filling; (iii) and selective systematic errors associated with lack of nocturnal mixing and associated data screening procedures [43, 44, 48, 49].

The method of Flanagan et al. [48] was applied to estimate to the random uncertainty (E_r_). The assumption of this method is that fluxs measured under the similar micrometeorological conditions on consecutive days should be similar and their any differences are related to the random error of eddy covariance measurements. Firstly, data from the same half hour period of consecutive days were screened under the similar micrometeorological conditions, which were defined as that the difference of Rn was less than 75 W m^-2^, the difference of air temperature was less than 3°C, and the difference of average wind speed was less than 1 m s^-1^. Then, the random uncertainty was calculated based on the standard deviation (*σ*) of differences between measurements made on different days (x_1_ and x_2_) when ‘‘equivalent environmental conditions” occurred, as followed:

$$E_{r}=\frac{1}{\sqrt{2}}\times \sigma_{\left(x_{1}-x_{2}\right)}$$ (3)

The fully systematic bias could be estimated by the method suggested by Aurela et al. [49]. Firstly, when there were longer than 3 day gaps in the data set, carbon dioxide fluxes were interpolated with the meteorological parameters obtained by shifting the corresponding period by 3 days backwards and forwards, separately. Then, the fully systematic biases were calculated according to the differences between the interpolated fluxes with the meteorological parameters obtained by the original period and those obtained by the shifted periods [50].

In our study, the u*-thresholds determined using the Reichstein’s method had a value per year, and varied with years. The selective systematic errors was calculated by decreasing or increasing 20% of the u* thresholds every year, respectively [28]. Finally, the three types of errors were combined using the error accumulation principle (combing them in quadrature) [48]. The uncertainty results are shown in S4 Table.

### Definitions of carbon dioxide flux uptake or release peak values, and corresponding periods

#### Definitions of α, NEP_max_, CUP, and β, NEP_min_ and CRP

Annual NEP can be decomposed into a net CUP and two net carbon dioxide release periods (CRP) at the beginning of the year (CRP__begin_) and the end of the year (CRP__end_, Fig 1). The CUP was defined as the number of days between BDOY and EDOY, representing the first and last days of positive NEP in a year [16–18], respectively. The maximum daily NEP (NEP_max_) was defined as the maximum value of daily NEP during CUP, and the minimum daily NEP (NEP_min_begin_ or NEP_min_end_) was defined as the minimum value of daily NEP during the release period (CRP__begin_ or CRP__end_). α was defined as the ratio of actual carbon dioxide flux uptake to a hypothetical maximum carbon dioxide flux uptake, which was defined as the product of CUP × NEP_*max*_, and β was defined as the ratio of actual carbon release to a hypothetical maximum carbon release, which was defined as the product of CRP × NEP_*min*_ [18]. Note that the 10-day moving average was applied to determine BDOY, EDOY, NEP_max_, NEP_min_begin_ and NEP_min_end_. Above all, annual NEP can be expressed as a function of the six indicators including α, CUP, NEP_*max*_, β, CPR and NEP_*min*_, namely NEP = α × CUP × NEP_*max*_ +β× CPR × NEP_*min*_.

A perturbation analysis was used to separate the contributions of the six indicators to the interannual variability of NEP. The total differential form of annual NEP with respect to the six indicators can explained more than 97% of the variability of NEP, and the differentials of all parameters were approximated by the anomalies (Δ) of these variables [18]. Therefore, relative contributions of the six indicators to NEP anomalies can be calculated as follows:

$$\xi_{x}=\frac{\sum_{i}\frac{\partial NEP}{\partial x}dx_{i}\frac{\left|\Delta NEP_{i}\right|}{\Delta NEP_{i}}}{\sum_{i}\left|\Delta NEP_{i}\right|}$$ (4)

Where *i* means the year from 2005 to 2018; *x* indicates *α*, *β*, *CUP*, *CRP*, *NEP*_*max*_ and *NEP*_*min*_; ξ_x_ represents the relative contributions of x, and its positive sign reveals identical anomalies of the indicator with annual NEP, and vice versa.

#### Definitions for climatic and biotic variables

At our study site, maize was planted in spring and harvested in autumn, which was similar across years (S1 Table). The whole year was divided into winter (December–February), spring (March–May), summer (June–August), and autumn (September–November), and the climatological seasons were consistent with the maize growth stages, when planting and seeding was in spring, vegetative and reproductive growth was in summer, mature and harvest was in autumn. Crop development during a certain stage requires a certain accumulated temperature, which was defined as the effective accumulated temperature (Ta__ef_) [51], and was calculated for each stage as follows:

$$Ta_{\_ef}=\sum T-nB$$ (5)

where n was the duration of the growth stage in days, T was the mean temperature for each growth stage, B was the base temperature defined as 10°C based on a previous study [51].

Seasonal and annual values of carbon dioxide fluxes (NEP, GEP, and RE), climatic (PAR, PPT, VPD, Ta and Ta__ef_), soil (T_s_ at 5cm and SWC), and biotic (LAI and Resi) variables were calculated to determine seasonal climatic and biotic effects on the variability of NEP.

### Statistical analyses

If variables had a significant linear regression over time, annual anomalies were defined as the differences between annual values and regression values over time, based on the procedure of linear detrending. Otherwise, annual anomalies were considered to be differences between the annual and long-term average values.

Correlation analysis was used to evaluate the influence of climatic and biotic variables on NEP, GEP, RE, NEP_max_\NEP_min_ and CUP\CRP. Structural equation modeling (SEM), a multivariate statistical method, was constructed to evaluate the direct or indirect effects of specific climate, soil, and vegetation variables (climatic and biotic controls) on annual anomalies in NEP by introducing intermediate variables (GEP and RE, and the NEP_max_\NEP_min_ and CUP\CRP) [52]. BDOY and EDOY which define the CUP, also were considered as the intermediate variables in the SEM. At first, the hypothesized causal relationships among variables were built based on prior knowledge of how climate, soil, and vegetation variables affect the annual anomalies of NEP. Then, we fitted the hypothesized model with the measured data, and removed the non-significant relationships with a stepwise regression. The final model that best fit our data was selected, and SEM models were evaluated using the Chi square test (χ^2^, p>0.05). The goodness of fit index (GFI > 0.8) and root mean square error of approximation (RMSEA < 0.05) were also used as references to evaluate the model [52].

Linear and stepwise regression were performed in SPSS 18.0 for Windows Software (Version 18.0, SPSS Inc., Chicago, IL, USA), and the significance level for the tests was p = 0.05. SEM analyses were done with AMOS 22.0 (Amos Development Corporation, Chicago, IL, USA).

## Results

### Trends in NEP, GEP, RE, NEP_max_, NEP_min_, CUP, and CRP, and their correlations

**Fig 2:**
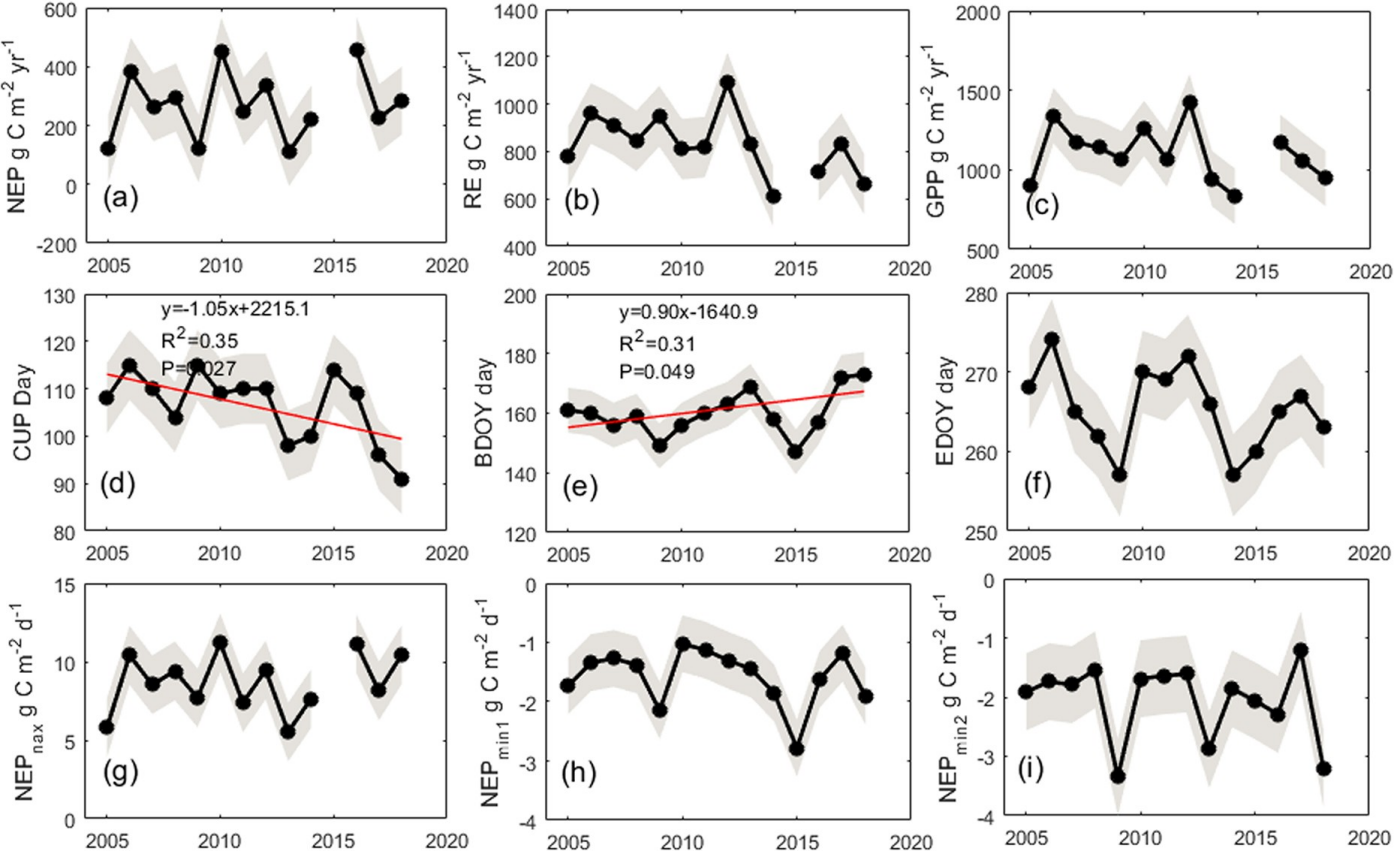
Annual values of net ecosystem production (NEP, a), ecosystem respiration (RE, b), gross ecosystem production (GEP, c), carbon dioxide uptake period (CUP, d), the beginning (BDOY, e) and ending date (EDOY, f) of net carbon dioxide uptake, maximum daily net ecosystem production (NEP_max_, g), and minimum daily net ecosystem production (NEP_min_begin,_ h, and NEP_min_end_, i). Red line indicates that there was a significant linear regression over time. Grey shading indicates standard deviation.

The annual average NEP, RE, and GEP was 270±31, 832±58, and 1102±86 (average ±uncertainty) g C m^−2^yr ^−1^ (S4 Table), respectively, and no significant increasing or decreasing trends were observed, although large interannual variations were present (Fig 2 and S2 Fig). Notably, CUP had a significant declining trend at 1.2 days per year, while NEP_max_ had no significant change trend over time. In addition, CRP had an increase trend, which equal to 365 –CUP, while NEP_min_ had no significant change trend over time. Annual values of NEP were significantly positively correlated with NEP_max_ (R^2^ = 0.85, P<0.001), while annual anomalies in NEP were positively correlated with CUP (R^2^ = 0.36, P = 0.04) and negatively with CRP at the beginning of the year (S5 and S6 Tables).

### Controlling factors of the interannual variability in NEP via GEP and RE

**Fig 3:**
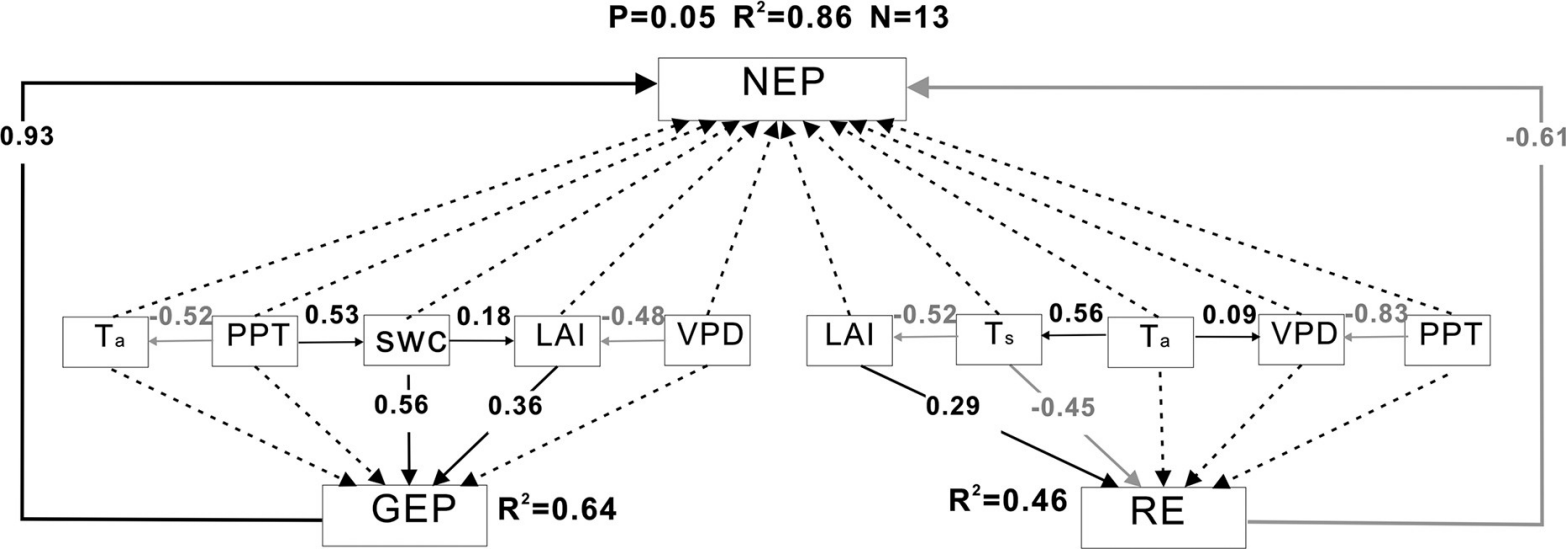
Results of the structural equation modeling (SEM) of the relationships among annual anomalies of climatic and biotic variables, gross ecosystem productivity (GEP), ecosystem respiration (RE), and net ecosystem production (NEP). Black arrows indicate significant positive relationships while gray arrows indicate significant negative relationships (P < 0.05). Black dashed arrows indicate insignificant relationships (P > 0.05). Numbers adjacent to arrows are path coefficients indicating the effect size for the relationship. The proportion of variance explained is given by R^2^.

SEM results showed that climatic and biotic variables acting via GEP and RE explained 86% of the variance in annual anomalies in NEP (Fig 3). The standardized direct effects of GEP and RE on annual anomalies in NEP were 0.93 and -0.61, respectively. SWC was the main controlling factor for GEP, while Ts was the main controlling factor for RE. GEP and RE also had positive correlations with LAI which was controlled by VPD, SWC and Ts. The standardized indirect effects of SWC, Ts and LAI on annual anomalies in NEP were 0.55, 0.19 and 0.16, respectively. VPD, PPT and Ta also indirectly affected annual anomalies in NEP by controlling SWC, Ts, and LAI and their standardized indirect effects on annual anomalies in NEP were -0.08, 0.31and 0.10, respectively.

### Controlling factors of the interannual variability in NEP via carbon dioxide uptake or release peak value and their periods

#### Influences of CUP and NEP_max_ during the year and the uptake period

**Fig 4:**
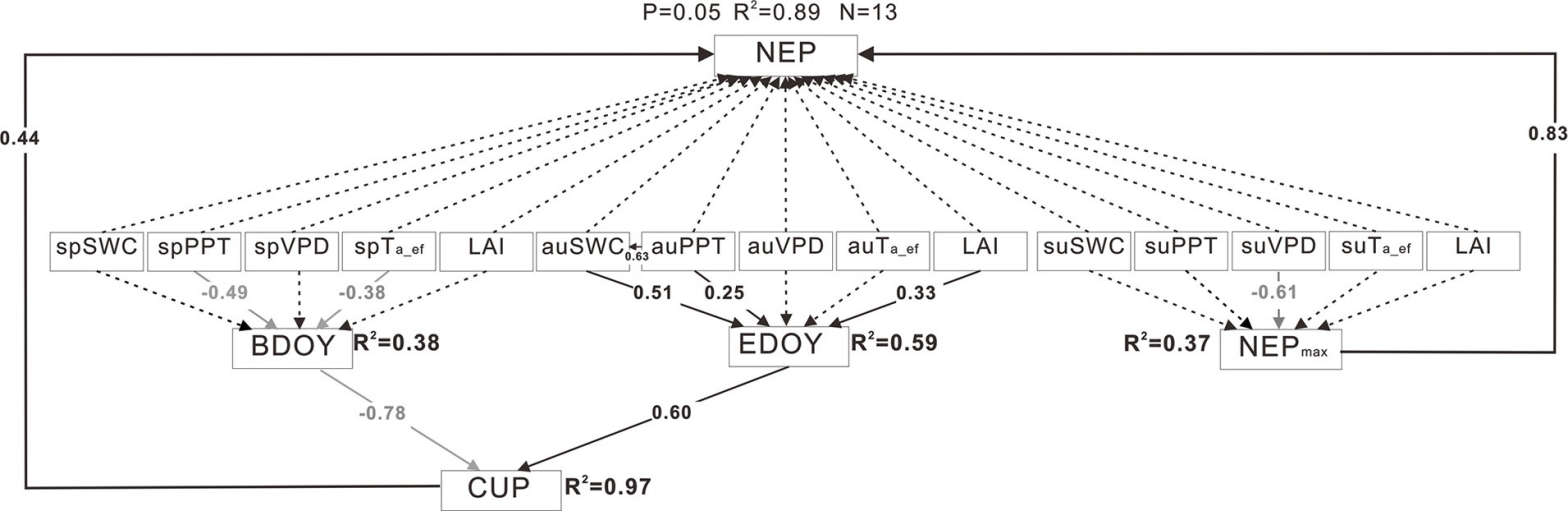
Results of structural equation modeling (SEM) of the relationships among annual anomalies of climatic and biotic variables, carbon dioxide uptake period (CUP) and maximum daily net ecosystem production (NEP_max_) and net ecosystem production (NEP) in an individual year. Black arrows indicate significant positive relationships, while gray arrows indicate significant negative relationships (P < 0.05). Black dashed arrows indicate insignificant relationships (P > 0.05). Numbers adjacent to arrows are path coefficients indicating the effect size of the relationship. The proportion of variance explained is given by R^2^. sp, su, and au indicate spring, summer, and autumn, respectively.

SEM analysis showed that CUP and NEP_max_ explained 89% of the variance in annual anomalies in NEP for the year (Fig 4), and 91% for the carbon dioxide uptake period (S3 Fig). The standardized direct effects of CUP and NEP_max_ on annual anomalies in NEP were 0.44 and 0.83 for the year, and 0.10 and 0.95 for CUP. The relative contributions of CUP, NEP_max_ and α to annual anomalies in NEP during the uptake period were 9.7, 84.1, and −3.1% (Table 1), respectively. Summer VPD affected on annual anomalies in NEP indirectly through its role in NEP_max_ with the standardized indirect effects of -0.51. BDOY was mainly controlled by spring precipitation and the effective accumulated temperature, and EDOY was mainly controlled by autumn precipitation, SWC and LAI. The standardized indirect effects of spring precipitation, spring effective accumulated temperature, autumn precipitation, autumn SWC and autumn LAI on annual anomalies in NEP ranged from 0.09 to 0.17. The effect of spring SWC on BDOY was not significant because SWC in spring was 0.08–0.18 cm^3^ cm^-3^ without waterlogging from 2005–2018.

**Table 1:** Relative contributions of the ratio of actual carbon dioxide uptake to hypothetical maximum carbon dioxide uptake (α), the maximum daily net ecosystem production (NEP_max_), the net carbon dioxide uptake period (CUP), the ratio of actual carbon release to hypothetical maximum carbon release (β), the minimum daily NEP (NEP_min_) and carbon release periods (CRP) to annual anomalies of net ecosystem production (NEP).

period		Parameters	contribution %
CUP		α	-3.1
		NEP_max_	84.1
		CUP	9.7
CRP	CRP__begin_	β__begin_	0.5
		NEP_min_begin_	2.3
		CRP__begin_	-1.5
	CRP__end_	β__end_	0.2
		NEP_min_end_	2.6
		CRP__end_	-0.9

#### Influences of CRP and NEP_min_ during the release period

**Fig 5:**
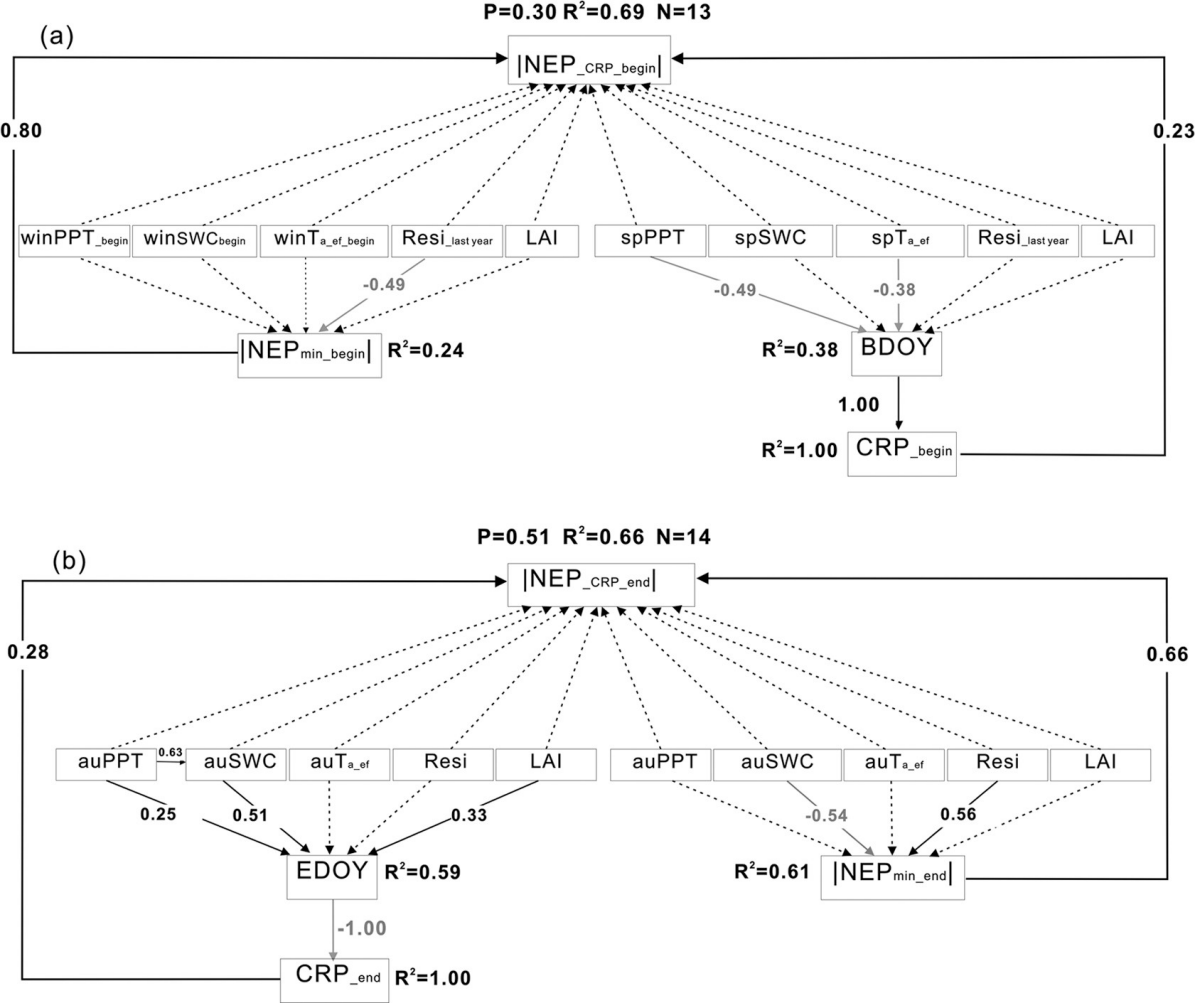
Results of the structural equation modeling of the relationships among annual anomalies of climatic and biotic variables, carbon dioxide release periods (CRP), minimum daily net ecosystem production (NEP_min_) and net ecosystem production (NEP) during the release period, (a) at the beginning of the year, and (b) at the end of the year. Black arrows indicate significant positive relationships, while gray arrows indicate significant negative relationships (P<0.05). Black dashed arrows indicate insignificant relationships (P>0.05). Numbers adjacent to arrows are path coefficients indicating the effect size of the relationship. The proportion of variance explained is given by R^2^. sp, au, and win indicate spring, autumn, and winter, respectively. Resi__last year_ indicates the amount of harvest residues remaining on the ground the previous year; Resi indicates the amount of residues in current year.

Climatic and biotic variables acting via CRP__begin_ and NEP_min_begin_ explained 69% of the variance in annual anomalies in carbon dioxide release amount at the beginning of the year (Fig 5A). The variance in annual anomalies in the carbon dioxide release amount at the beginning of the year were mainly driven by NEP_min_ at the beginning of the year, which was mainly restricted by the residues cover of the previous year. The amount of carbon dioxide release was also positively correlated with CRP at the beginning of the year (R^2^ = 0.33, P = 0.05), and this was mainly controlled by spring PPT and Ta through BDOY (R^2^ = 0.38). However, the influence of NEP_min_ at the beginning of the year was dominant, and the relative contributions of CRP_,_ NEP_min_ and β to annual anomalies in NEP were 0.5, 2.3, and −1.5% (Table 1), respectively.

Climatic and biotic variables acting via the CRP__end_ and NEP_min_end_ explained 66% of the variance in annual anomalies in carbon dioxide release amount at the end of the year (Fig 5B). The variance in annual anomalies in carbon dioxide release amount at the end of the year were mainly driven by NEP _min_ at the end of the year with the standardized direct effects 0.66. NEP _min_ at the end of the year increased by the left residues of the current year and restricted by SWC (Fig 5B). The standardized direct effects of CRP at the end of the year on the variance in annual anomalies in carbon dioxide release amount were 0.28 and lower than 0.66 of NEP_min_end_. The CRP at the end of the year was mainly controlled by autumn SWC, LAI, and autumn PPT acting via EDOY (Fig 5B). The relative contributions of CRP_,_ NEP_min_ and β at the end of the year for annual anomalies in NEP were 0.2, 2.6 and −0.9% (Table 1), respectively.

## Discussion

### Partitioning of NEP into GEP and RE, and carbon dioxide uptake or release peak value and their periods

The effects on the interannual variability in NEP of GEP that was mainly controlled by soil water content were more than those of RE that was mainly controlled by temperature in a maize agroecosystem in northeastern China. Deficits in precipitation lead to a decrease in soil moisture causing stomatal closure or directly affecting plant growth, and further reducing photosynthesis [53]. Increasing temperature can directly stimulate enzyme activity and accelerate both photosynthesis and respiration rates. However, too high temperature is often accompanied by drought and water deficit can lead to the decreasing of photosynthesis and respiration [17]. The influence of GEP on interannual variability in NEP was greater than that of RE, confirming results derived from published literature on 20 sites and different farmlands at a regional scale [4]. These indicated that water conditions (e.g. PPT, SWC and VPD) were the dominant factor in interannual variability in NEP for this agroecosystem, while temperature may be an important indirect factor when VPD is dominated.

The effects on the interannual variability in NEP of NEP_max_ that was controlled by summer VPD was more than those of CUP that was controlled by PPT. The shortage of moisture (an increase in VPD) in summers can limit plant growth, which was indicated as a physiological factor NEP_max_ [54, 55]. Spring precipitation and the effective accumulated temperature affected on interannual variability in NEP indirectly through their roles in BDOY, and autumn precipitation, SWC and LAI affected on annual anomalies in NEP indirectly through their roles in EDOY. The increase in spring precipitation and the effective accumulated temperature can lead to advancement of vegetation phenology [23]. Autumn phenology was dominated by autumn SWC, and soil water supply enabled carbon gain longer in the autumn [56]. The advancement of BDOY and delay of EDOY increase the length of the growing season in warmer and wetter years, and that affects the cumulative sum of assimilated carbon and NEP [17].

The effects on the interannual variability in NEP during the carbon dioxide release period of NEP_min_begin_ that was controlled by residues cover of previous year, was more than those of CRP__begin_ that was controlled by spring PPT and T_a_ef_ at the beginning of the year. Although soil temperature was highly correlated to air temperature (y = 0.65x, R^2^ = 0.78, P <0.001) in winter, the correlation between the two parameters in spring decreased (y = 1.25x-0.02, R^2^ = 0.55, P <0.001). Residues cover from a previous year might inhibit the increases in soil temperatures in spring (y = -0.01x+7.72, R^2^ = 0.34, P <0.001, S4 Fig), and reduce soil respiration rates [56]. Vegetation phenology advance with the increasing spring precipitation and effective accumulated air temperature can lead to a decrease in CRP__begin_. However, soil respiration was less sensitive to spring precipitation and temperature, due to a cover of organic residues on the soil at a no-till site [56].

The effects on the interannual variability in NEP during the carbon dioxide release period of NEP_min_end_ that was controlled by current residues and autumn SWC, was also more than those of CRP__end_ that was controlled by autumn PPT, LAI and SWC at the end of the year. The existing research found there was a positive linear relationship between harvest residue amounts and basal respiration rate at 10°C, indicating that residues supplied materials for respiration and promoted carbon release [28].

### Trends in the interannual variability in NEP and implications for agricultural practices

In our study, there was no significant increasing or decreasing trend in NEP, because interannual variability in NEP was predominately determined by NEP_max_ which exhibited no significant change trend; however, CUP had a significant declining trend of 1.2 days per year. Climatic factors including PAR and VPD had a notably increasing trend (S5 Fig). However, the increasing light can increase VPD by rising temperature, and enhance the inhibiting effect of increasing VPD on NEP exerted by GEP and NEP_max_ (S6 Fig). In addition, precipitation is expected to decrease by 13.3 mm/10a in the agroecosystems of northeastern China, as indicated by ground observations at 91 meteorological stations for years 1961–2010 [29]. A declining precipitation can result in a decrease in NEP by decreasing GEP, NEP_max_, and CUP. Spring precipitation is the main driver of the variability in BDOY, with an average delay of 0.08 days per millimeter decrease in precipitation; a delay in BDOY can decrease CUP and lead to a decrease in NEP. In our study, a delay in BDOY increasing at 0.9 days per year and a decrease in CUP has been observed (Fig 2) due to a decrease in spring precipitation at 3 mm per year. Thus, NEP may decrease in response to a declining precipitation of climate change.

Some agricultural management practices may be useful in efforts to modulate the response of NEP to climate change; among them, irrigation or use of organic residues appears beneficial. In our study, only the grains were removed from the field, and thus NBP was caculated by the diffference between NEP and the carbon amount of grains. The average NBP from 2005 to 2018 was 11±124 g C m^-2^ y^-1^ (mean ± standard deviation), and there were no significant differences among the multi-year NBP (P>0.05), indicating that the study site was carbon neutral. The positive correlatation between NBP and NEP showed that the increasing NEP can really result in the increasing of carbon uptake (R^2^ = 0.91, P<0.001). Adequate soil moisure was important in determining the best time to plant [2], and planting date may be strategically delayed to avoid water stress. BDOY might be moved backward with planting date deferment. Planting usually occurs after the first rain event > 10 mm in our study area in northeastern China [37]. Irrigation can be an effective crop management method to increase carbon dioxide uptake period by advancing BDOY and promoting plant growth in summer. In addition, presence of harvest residues limited carbon dioxide release at the beginning of the year, and stimulated it at the end of the year. However, not-till management can reduce carbon dioxide release.

## Conclusions

Our results showed that the rain-fed spring maize ecosystem in northeastern China acted as a carbon dioxide sink from 2005 to 2018, and the annual NEP was 270±31 g C m^−2^yr ^−1^ without a change over time. GEP, RE, and NEP_max_ also exhibited no signifcant change trend, although CUP showed a significant decline trend. The effects on the interannual variability in NEP of GEP that was controlled by soil water content was more than those of RE that was controlled by temperature. Further, the effects on the interannual variability in NEP of NEP_max_ that was controlled by summer VPD were more than those of CUP that was defined by BDOY and EDOY. BDOY was mainly affected by spring precipitation and the effective accumulated temperature, and EDOY was affected by autumn precipitation, SWC and LAI. However, carbon dioxide uptake was the dominant component of interannual variability in NEP, and water conditions (e.g. PPT, SWC and VPD) were the dominant factor of interannual variability in NEP, especially moisture in summer. In response to climate change with a declining precipitation, NEP may decrease in the future, and irrigation or residues cover may be useful for alleviating the effect. Overall, Our results indicate that interannual variability in NEP in agroecosystems may be more sensitive to changes in water conditions (such as precipitation, SWC and VPD) induced by climate changes, while temperature may be an important indirect factor when VPD is dominated.

## Supporting information

S1 Table The growth stage of maize from 2005 to 2018 in Jinzhou. Rain-fed maize is generally sown every year during mid-April to mid-May without rotation, and harvested during mid-September to early October.
(DOCX)

S2 Table The correlation between LAI and NDVI during each maize growth stage from 2005 to 2018. Daily LAI was used as a plant variables, and NDVI (16-day) from MODIS image was obtained for building the correlation between LAI and NDVI during each maize growth stage, and then getting daily LAI by linearly interpolating the 16-day LAI.
(DOCX)

S3 Table The proportion of effective data after quality control during 2005–2018. Data classified with flag 2 were removed. When friction velocity (u*) was < the u*-threshold, flux data were also rejected to avoid possible underestimation of flux during stable conditions at night. The u*-threshold was determined using the Reichstein et al. method.
(DOCX)

S4 Table Annual values of net ecosystem production (NEP), ecosystem respiration (RE), gross ecosystem production (GEP), carbon dioxide flux uptake period (CUP), the beginning and ending date of net carbon dioxide flux uptake (BDOY and EDOY), maximum daily net ecosystem production (NEP_max_), and minimum daily NEP (NEP_min_begin_ and NEP_min_end_). The Annual values of net ecosystem production and relative parameters were presented from 2005 to 2018. Plus/minus values were the estimates of uncertainties, and SD means standard deviation.
(DOCX)

S5 Table Linear regressions among annual values and anomalies of net ecosystem production (NEP), gross ecosystem productivity (GEP) and ecosystem respiration (RE). The Linear relationships between annual values and anomalies of NEP and GEP (and RE) were presented. ** and * represent a significant relationship at p = 0.01, and 0.05 levels, respectively.
(DOCX)

S6 Table Linear regressions between annual values and anomalies of net ecosystem production (NEP), and that of the carbon dioxide flux uptake and release peak value (NEP_max_, NEP_min_begin_ and NEP_min_end_) and corresponding periods (CUP, CRP__begin_ and CRP__end_). The Linear relationships between annual values and anomalies of NEP and carbon uptake and release peak values (and periods) were presented. ***, ** and * represent a significant relationship at p = 0.001, 0.01, and 0.05 levels, respectively.
(DOCX)

S1 Fig Flux footprint climatology maps (a) and wind direction map (b). Black contour lines of (a) indicat flux contribution from 90% (outer) to 10% (inner), with 10% intervals.
(TIF)

S2 Fig Seasonal and annual dynamics of daily net ecosystem production (NEP, a), gross ecosystem production (GEP, b) and ecosystem respiration (RE, c) from 2005 to 2018. Gray lines indicate 10-day moving average NEP, black triangle indicates maximum daily net ecosystem production (NEP_max_), black circles indicate the beginning and ending date of net carbon uptake (BDOY and EDOY), black points indicate minimum daily NEP (NEP_min_).
(TIF)

S3 Fig The structure equation modeling results of the relationship among annual anomalies of climatic and biotic variables, carbon uptake period (CUP) and maximum daily net ecosystem production (NEP_max_) and net ecosystem production (NEP) during uptake period. Black arrows indicate significant positive relationships while gray arrows indicate significant negative relationships (P < 0.05). Black dashed arrows indicate insignificant relationships (P > 0.05). Numbers adjacent to arrows are path coefficients and indicative of the effect size of the relationship. The proportion of variance explained (R^2^) appears alongside every response variable in the model. sp, su, and au indicate spring, summer, and autumn, respectively.
(TIF)

S4 Fig Dependence of annual average soil temperature from DOY 1 to planting date on the residues of last year. Grey area was 95% confidence limits. Grey dotted lines were prediction bounds. Black line was the fitted curve.
(TIF)

S5 Fig Annual values of PAR(a), PPT(b), Ta(c), VPD(d), Ts(e), SWC(f), LAI(g) and Resi(h). The red line indicate that there was a significant linear regression with time. Grey area was 95% confidence limits.
(TIF)

S6 Fig The structure equation modeling results of the relationship between annual anomalies of climatic variables and net ecosystem production (NEP). Black arrows indicate significant positive relationships while gray arrows indicate significant negative relationships (P < 0.05). Numbers adjacent to arrows are path coefficients and indicative of the effect size of the relationship. The proportion of variance explained (R^2^) appears alongside every response variable in the model.
(TIF)
