# Supplementary material for: Interannual variability in net ecosystem carbon production in a rain-fed maize ecosystem and its climatic and biotic controls during 2005–2018
Source: PLoS One. 2021 May 10;16(5):e0237684. doi: 10.1371/journal.pone.0237684 (PMC8109796; doi:10.1371/journal.pone.0237684)
Supplement: S1 Table — Rain-fed maize is generally sown every year during mid-April to mid-May without rotation, and harvested during mid-September to early October. (DOCX) [file pone.0237684.s001.docx]

**S1 Table. The growth stage of maize from 2005 to 2018 in Jinzhou.** SD means standard deviation.

| **year** | **Plant** | **Germinate** | **Three leaves** | **Seven leaves** | **Jointing** | **Tasseling** | **Filling** | **Mature** | **Harvest** |
| --- | --- | --- | --- | --- | --- | --- | --- | --- | --- |
| 2005 | 1 May | 14 May | 21 May | 11 Jun | 23 Jun | 19 Jul | 23 Aug | 26 Sep | 1 Oct |
| 2006 | 4 May | 18 May | 24 May | 8 Jun | 24 Jun | 19 Jul | 20 Aug | 27 Sep | 2 Oct |
| 2007 | 3 May | 10 May | 17 May | 1 Jun | 20 Jun | 16 Jul | 15 Aug | 24 Sep | 2 Oct |
| 2008 | 27 Apr | 9 May | 15 May | 5 Jun | 25 Jun | 21 Jul | 17 Aug | 25 Sep | 2 Oct |
| 2009 | 27 Apr | 10 May | 16 May | 31 May | 20 Jun | 16 Jul | 14 Aug | 22 Sep | 4 Oct |
| 2010 | 2 May | 17 May | 22 May | 6 Jun | 24 Jun | 19 Jul | 18 Aug | 28 Sep | 3 Oct |
| 2011 | 3 May | 15 May | 21 May | 5 Jun | 20 Jun | 19 Jul | 18 Aug | 26 Sep | 1 Oct |
| 2012 | 11 May | 23 May | 28 May | 13 Jun | 29 Jun | 22 Jul | 15 Aug | 29 Sep | 4 Oct |
| 2013 | 10 May | 20 May | 26 May | 7 Jun | 23 Jun | 24 Jul | 18 Aug | 23 Sep | 29 Sep |
| 2014 | 9 May | 19 May | 23 May | 7 Jun | 19 Jun | 23 Jul | 15 Aug | 16 Sep | 29 Sep |
| 2015 | 20 Apr | 5 May | 10 May | 27 May | 12 Jun | 12 Jul | 20 Aug | 21 Sep | 26 Sep |
| 2016 | 5 May | 17 May | 20 May | 27 May | 17 Jun | 15 Jul | 19 Aug | 20 Sep | 27 Sep |
| 2017 | 21 May | 29 May | 31 May | 7 Jun | 28 Jun | 23 Jul | 23 Aug | 23 Sep | 30 Sep |
| 2018 | 23 May | 29 May | 1 Jun | 11 Jun | 23 Jun | 28 Jul | 27 Aug | 3 Oct | 5 Oct |
| mean | 5 May | 16 May | 21 May | 5 Jun | 21 Jun | 19 Jul | 18 Aug | 24 Sep | 1 Oct |
| SD | 9 | 7 | 6 | 5 | 4 | 4 | 4 | 4 | 3 |
