# Supplementary material for: Interannual variability in net ecosystem carbon production in a rain-fed maize ecosystem and its climatic and biotic controls during 2005–2018
Source: PLoS One. 2021 May 10;16(5):e0237684. doi: 10.1371/journal.pone.0237684 (PMC8109796; doi:10.1371/journal.pone.0237684)
Supplement: S2 Table — Daily LAI was used as a plant variables, and NDVI (16-day) from MODIS image was obtained for building the correlation between LAI and NDVI during each maize growth stage, and then getting daily LAI by linearly interpolating the 16-day LAI. (DOCX) [file pone.0237684.s002.docx]

**S2 Table. The correlation between leaf area index (x) and NDVI (y) during each maize growth stage from 2005 to 2018.**

| Year | Correlation | R^2^ | P |
| --- | --- | --- | --- |
| 2005 | y=0.0006e^12.08x^ | 0.96 | 0.029 |
| 2006 | y=10.79x - 3.17 | 0.93 | 0.032 |
| 2007 | y=8.05x - 2.7 | 0.92 | 0.040 |
| 2008 | y=7.27x - 1.98 | 0.91 | 0.043 |
| 2009 | y=0.0003e^14.37x^ | 0.99 | 0.003 |
| 2010 | y=9.73x - 2.80 | 0.92 | 0.044 |
| 2011 | y=0.0008e^12.17x^ | 0.88 | 0.025 |
| 2012 | y=0.0009e^12.10x^ | 0.87 | 0.018 |
| 2013 | y=9.94x - 3.18 | 0.92 | 0.001 |
| 2014 | y=0.0003e^14.54x^ | 0.96 | 0.015 |
| 2015 | y=0.0015e^13.58x^ | 0.99 | 0.008 |
| 2016 | y=0.0085e^9.13x^ | 0.99 | 0.013 |
| 2017 | y=11.54x - 3.15 | 0.97 | 0.006 |
| 2018 | y=7.71x - 2.07 | 0.98 | 0.009 |
