# Supplementary material for: Interannual variability in net ecosystem carbon production in a rain-fed maize ecosystem and its climatic and biotic controls during 2005–2018
Source: PLoS One. 2021 May 10;16(5):e0237684. doi: 10.1371/journal.pone.0237684 (PMC8109796; doi:10.1371/journal.pone.0237684)
Supplement: S3 Table — Data classified with flag 2 were removed. When friction velocity (u*) was < the u*-threshold, flux data were also rejected to avoid possible underestimation of flux during stable conditions at night. The u*-threshold was determined using the Reichstein et al. method. (DOCX) [file pone.0237684.s003.docx]

**S3 Table. The proportion of effective data after quality control during 2005-2018.**

| year | After Flag 2 removed % | After u* threshold removed % | day % | night % |
| --- | --- | --- | --- | --- |
| 2005 | 57.8 | 47.5 | 67.8 | 26.3 |
| 2006 | 61.1 | 50.2 | 75.4 | 24.2 |
| 2007 | 45.1 | 39.0 | 62.2 | 15.5 |
| 2008 | 50.0 | 40.9 | 66.5 | 15.2 |
| 2009 | 54.0 | 44.1 | 67.8 | 20.3 |
| 2010 | 50.2 | 43.4 | 67.2 | 15.5 |
| 2011 | 49.4 | 41.8 | 67.9 | 15.5 |
| 2012 | 54.9 | 45.1 | 73.9 | 14.6 |
| 2013 | 57.2 | 46.5 | 74.9 | 17.9 |
| 2014 | 59.0 | 45.7 | 73.7 | 19.5 |
| 2015 | 24.3 | 18.1 | 25.8 | 12.9 |
| 2016 | 58.7 | 44.6 | 59.3 | 29.2 |
| 2017 | 54.2 | 48.1 | 65.1 | 30.3 |
| 2018 | 58.8 | 45.1 | 66.3 | 35.3 |
