# Supplementary material for: Interannual variability in net ecosystem carbon production in a rain-fed maize ecosystem and its climatic and biotic controls during 2005–2018
Source: PLoS One. 2021 May 10;16(5):e0237684. doi: 10.1371/journal.pone.0237684 (PMC8109796; doi:10.1371/journal.pone.0237684)
Supplement: S4 Table — The Annual values of net ecosystem production and relative parameters were presented from 2005 to 2018. Plus/minus values were the estimates of uncertainties, and SD means standard deviation. (DOCX) [file pone.0237684.s004.docx]

**S4 Table. Annual values of net ecosystem production (NEP), ecosystem respiration (RE), gross ecosystem production (GEP), carbon dioxide flux uptake period (CUP), the beginning and ending date of net carbon dioxide flux uptake (BDOY and EDOY), maximum daily net ecosystem production (NEP_max_), and minimum daily NEP (NEP_min_begin_ and NEP_min_end_).** Plus/minus values are the estimates of uncertainties, and SD means standard deviation.

| year | NEP | RE | GEP | CUP | BDOY | EDOY | NEP_max_ | NEP_min_begin_ | NEP_min_end_ |
| --- | --- | --- | --- | --- | --- | --- | --- | --- | --- |
|  | gCm^-2^yr^-1^ | gCm^-2^yr^-1^ | gCm^-2^yr^-1^ | doy | doy | doy | gCm^-2^d^-1^ | gCm^-2^d^-1^ | gCm^-2^d^-1^ |
| 2005 | 122±13 | 778±49 | 901±53 | 108 | 161 | 268 | 5.79 | -1.73 | -1.91 |
| 2006 | 381±36 | 960±54 | 1342±72 | 115 | 160 | 274 | 10.41 | -1.34 | -1.74 |
| 2007 | 261±23 | 912±63 | 1173±90 | 110 | 156 | 265 | 8.54 | -1.27 | -1.79 |
| 2008 | 295±24 | 844±51 | 1139±78 | 104 | 159 | 262 | 9.41 | -1.38 | -1.54 |
| 2009 | 121±20 | 949±42 | 1069±68 | 109 | 149 | 257 | 7.7 | -2.15 | -3.35 |
| 2010 | 451±54 | 809±75 | 1260±92 | 115 | 156 | 270 | 11.2 | -1.02 | -1.69 |
| 2011 | 248±27 | 819±31 | 1067±63 | 110 | 160 | 269 | 7.39 | -1.13 | -1.64 |
| 2012 | 338±47 | 1089±64 | 1427±87 | 110 | 163 | 272 | 9.46 | -1.3 | -1.61 |
| 2013 | 109±17 | 834±55 | 944±76 | 98 | 169 | 266 | 5.53 | -1.45 | -2.88 |
| 2014 | 221±18 | 613±64 | 834±82 | 100 | 158 | 257 | 7.64 | -1.85 | -1.86 |
| 2015 | - | - | - | 114 | 147 | 260 | - | -1.78 | -1.06 |
| 2016 | 455±55 | 718±88 | 1172±101 | 109 | 157 | 265 | 11.14 | -1.62 | -2.29 |
| 2017 | 225±18 | 833±42 | 1058±67 | 96 | 172 | 267 | 8.14 | -1.18 | -1.21 |
| 2018 | 285±29 | 662±63 | 946±81 | 91 | 173 | 263 | 10.44 | -1.91 | -3.21 |
| mean | 270±31 | 832±58 | 1102±86 | 106 | 160 | 265 | 8.68 | -1.51 | -1.98 |
| SD | 115±23 | 128±35 | 175±43 | 7.42 | 7.54 | 5.18 | 1.87 | 0.34 | 0.70 |
