# Supplementary material for: Interannual variability in net ecosystem carbon production in a rain-fed maize ecosystem and its climatic and biotic controls during 2005–2018
Source: PLoS One. 2021 May 10;16(5):e0237684. doi: 10.1371/journal.pone.0237684 (PMC8109796; doi:10.1371/journal.pone.0237684)
Supplement: S5 Table — The Linear relationships between annual values and anomalies of NEP and GEP (and RE) were presented. ** and * represent a significant relationship at p = 0.01, and 0.05 levels, respectively. (DOCX) [file pone.0237684.s005.docx]

**S5 Table. Linear regressions among annual values and anomalies of net ecosystem production (NEP), gross ecosystem productivity (GEP) and ecosystem respiration (RE).** ^**^ and ^*^ represent a signiﬁcant relationship at p = 0.01, and 0.05 levels, respectively.

| Variables | Linear regressions | R^2^ | P |
| --- | --- | --- | --- |
| annual values | NEP=0.44×GEP-199.5 | 0.51 | **0.010^**^** |
|  | NEP=0.13×RE+175.9 | 0.02 | 0.931 |
|  | RE=0.56×GEP+199.4 | 0.64 | **0.003^**^** |
| annual anomalies | NEP__ anomalies_ =0.44×GEP__ anomalies_+1×10 ^13^ | 0.51 | **0.010^**^** |
|  | NEP__ anomalies_ =0.13×RE__ anomalies_ +2×10 ^14^ | 0.02 | 0.931 |
|  | RE__ anomalies_ =0.56×GEP__ anomalies_ +6×10 ^14^ | 0.64 | **0.003^**^** |
