# Supplementary material for: Interannual variability in net ecosystem carbon production in a rain-fed maize ecosystem and its climatic and biotic controls during 2005–2018
Source: PLoS One. 2021 May 10;16(5):e0237684. doi: 10.1371/journal.pone.0237684 (PMC8109796; doi:10.1371/journal.pone.0237684)
Supplement: S6 Table — The Linear relationships between annual values and anomalies of NEP and carbon uptake and release peak values (and periods) were presented. ***, ** and * represent a significant relationship at p = 0.001, 0.01, and 0.05 levels, respectively. (DOCX) [file pone.0237684.s006.docx]

**S6 Table. Linear regressions between annual values and anomalies of net ecosystem production (NEP), and that of the carbon dioxide flux uptake and release peak value (NEP_max_, NEP_min_begin_ and NEP_min_end_) and corresponding duration (CUP, CRP__begin_ and CRP__end_).** ^***^, ^**^ and ^*^ represent a signiﬁcant relationship at p=0.001, 0.01, and 0.05 levels, respectively.

| Variables (y) |  | Variables (x) | Linear regressions | R^2^ | P |
| --- | --- | --- | --- | --- | --- |
| annual values of NEP | uptake | CUP | y =6.59x - 426.85 | 0.18 | 0.149 |
|  |  | NEP_max_ | y =56.90x - 223.34 | 0.85 | **<0.001^***^** |
|  | release | CRP__begin_ | y =-2.28x + 636.61 | 0.19 | 0.661 |
|  |  | CRP__end_ | y =-8.84x + 1149.6 | 0.16 | 0.183 |
|  |  | NEP_min_begin_ | y =149.71x + 492.74 | 0.2 | 0.129 |
|  |  | NEP_min_end_ | y =60.586x + 394.76 | 0.13 | 0.236 |
| annual anomalies of NEP | uptake | CUP__aomalies_ | y =15.46x + 0.57 | 0.51 | **0.006^**^** |
|  |  | NEP_max_aomalies_ | y =56.90x + 2E-14 | 0.85 | **<0.001^***^** |
|  | release | CRP__begin _aomalies_ | y =-13.47x + 23.23 | 0.36 | **0.040^*^** |
|  |  | CRP__end_aomalies_ | y =-8.84x -6.02 | 0.16 | 0.183 |
|  |  | NEP_min_begin _aomalies_ | y =149.71x -13.83 | 0.2 | 0.129 |
|  |  | NEP_min_end_aomalies_ | y =60.59x + 1.28 | 0.13 | 0.236 |
