# Supplementary figures and images for: Interannual variability in net ecosystem carbon production in a rain-fed maize ecosystem and its climatic and biotic controls during 2005–2018
Source: PLoS One. 2021 May 10;16(5):e0237684. doi: 10.1371/journal.pone.0237684 (PMC8109796; doi:10.1371/journal.pone.0237684)

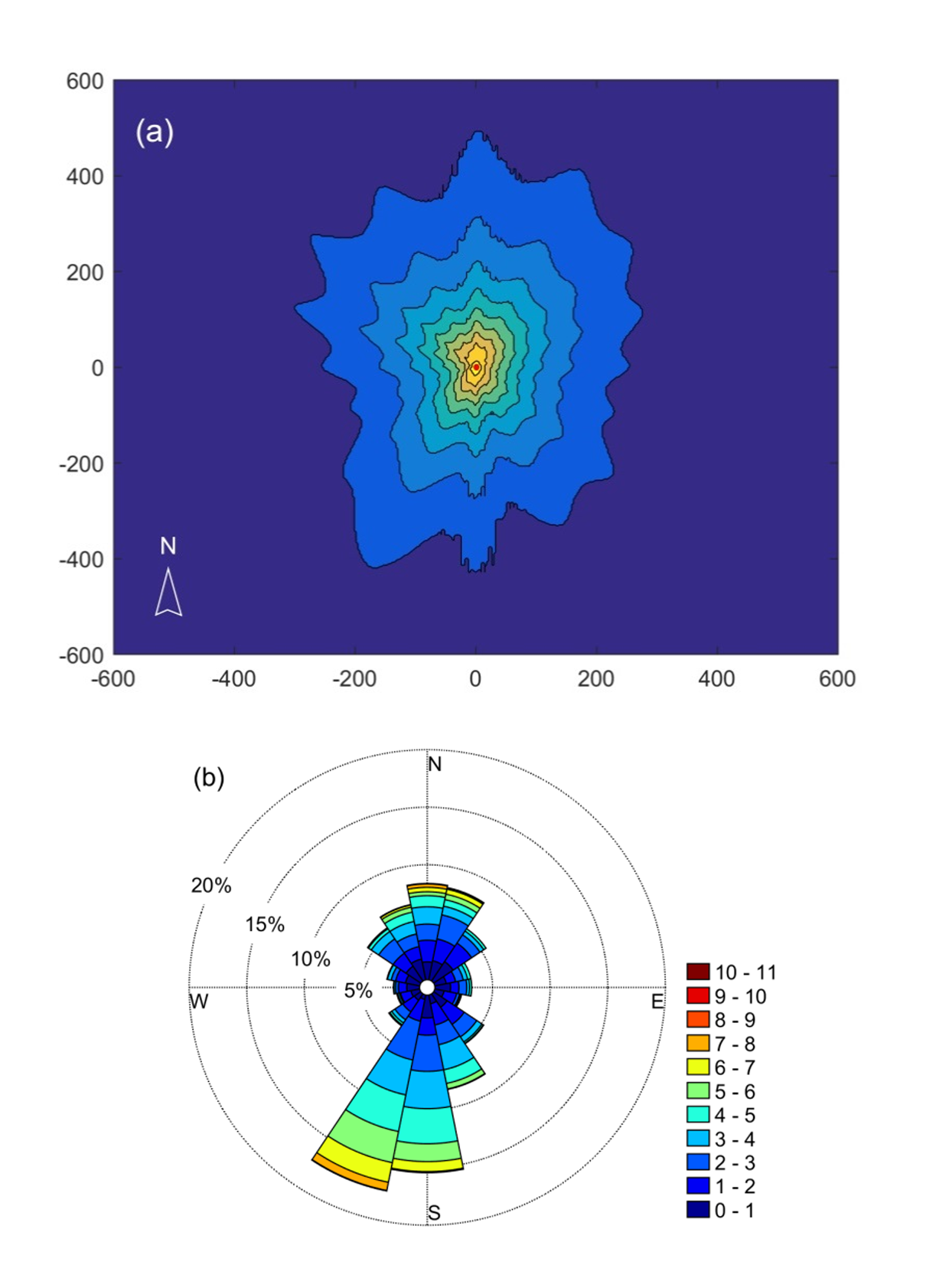

Supplement: S1 Fig — Flux footprint climatology maps (a) and wind direction map (b). Black contour lines of (a) indicat flux contribution from 90% (outer) to 10% (inner), with 10% intervals. (TIF) [file pone.0237684.s007.tif]

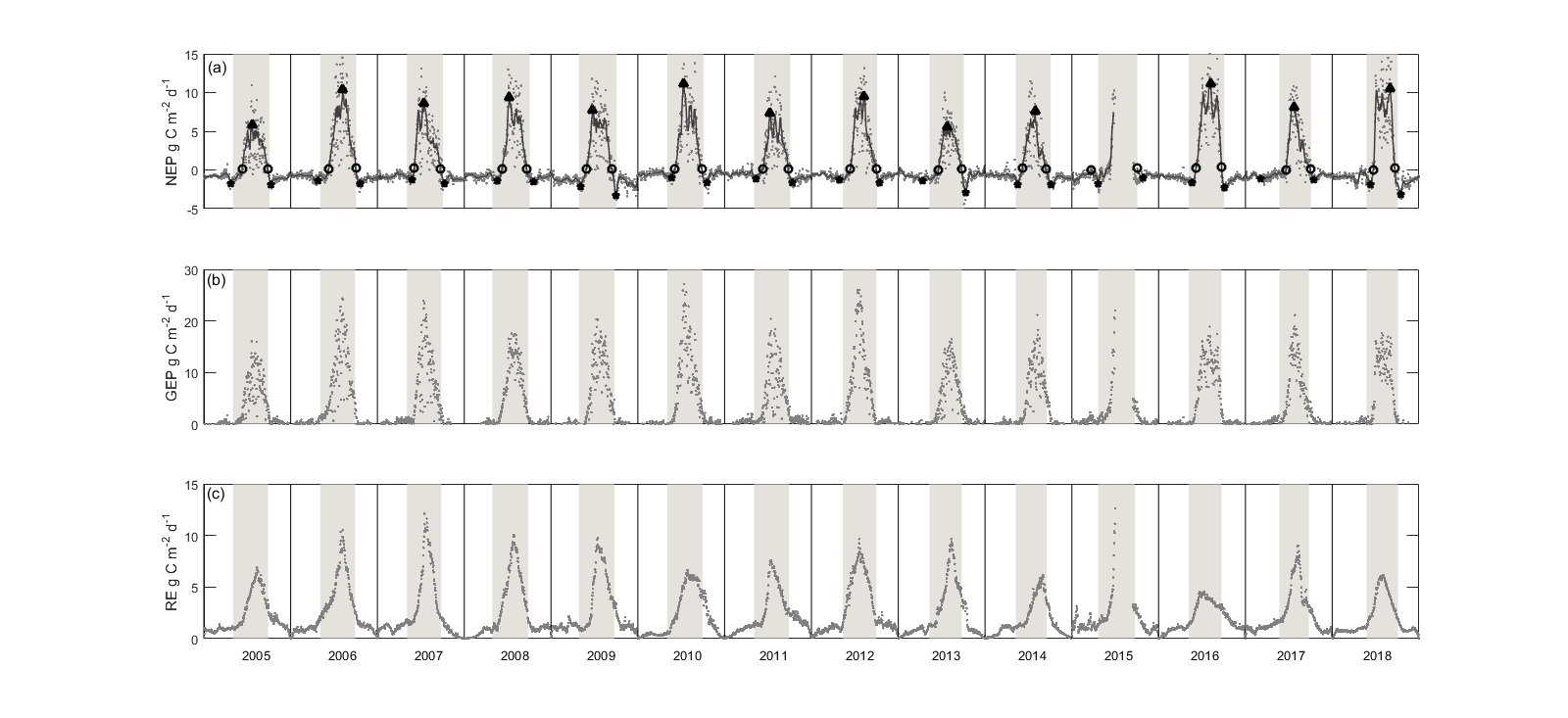

Supplement: S2 Fig — Seasonal and annual dynamics of daily net ecosystem production (NEP, a), gross ecosystem production (GEP, b) and ecosystem respiration (RE, c) from 2005 to 2018. Gray lines indicate 10-day moving average NEP, black triangle indicates maximum daily net ecosystem production (NEPmax), black circles indicate the beginning and ending date of net carbon uptake (BDOY and EDOY), black points indicate minimum daily NEP (NEPmin). (TIF) [file pone.0237684.s008.tif]

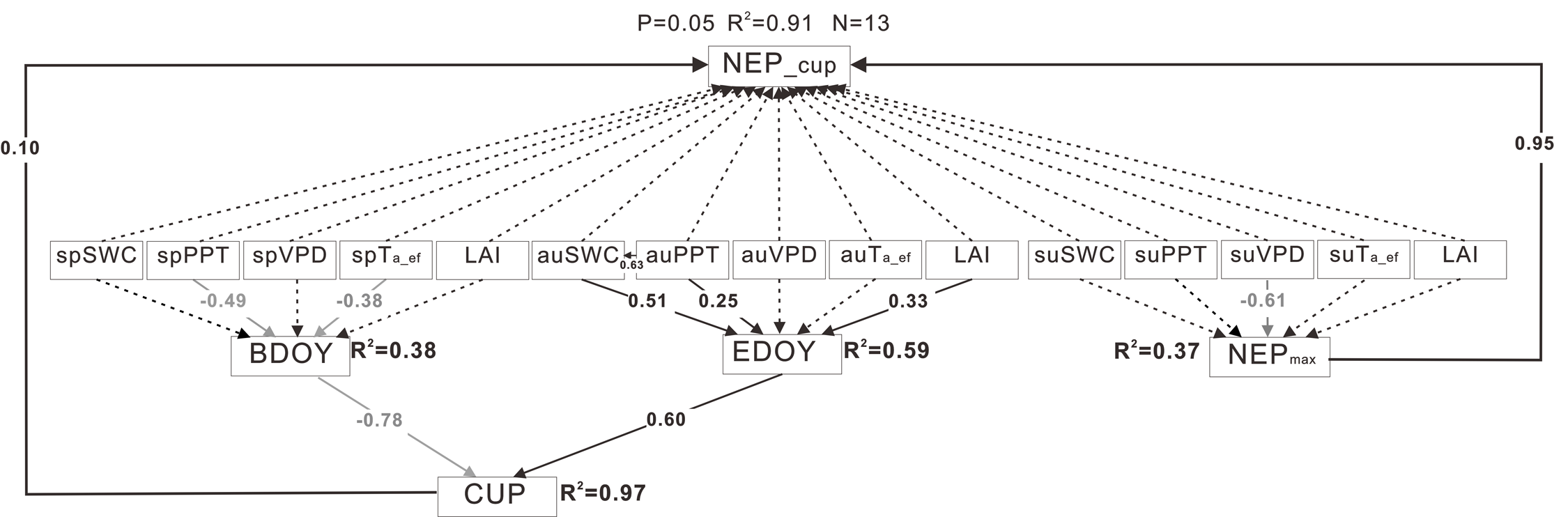

Supplement: S3 Fig — Black arrows indicate significant positive relationships while gray arrows indicate significant negative relationships (P < 0.05). Black dashed arrows indicate insignificant relationships (P > 0.05). Numbers adjacent to arrows are path coefficients and indicative of the effect size of the relationship. The proportion of variance explained (R2) appears alongside every response variable in the model. sp, su, and au indicate spring, summer, and autumn, respectively. (TIF) [file pone.0237684.s009.tif]

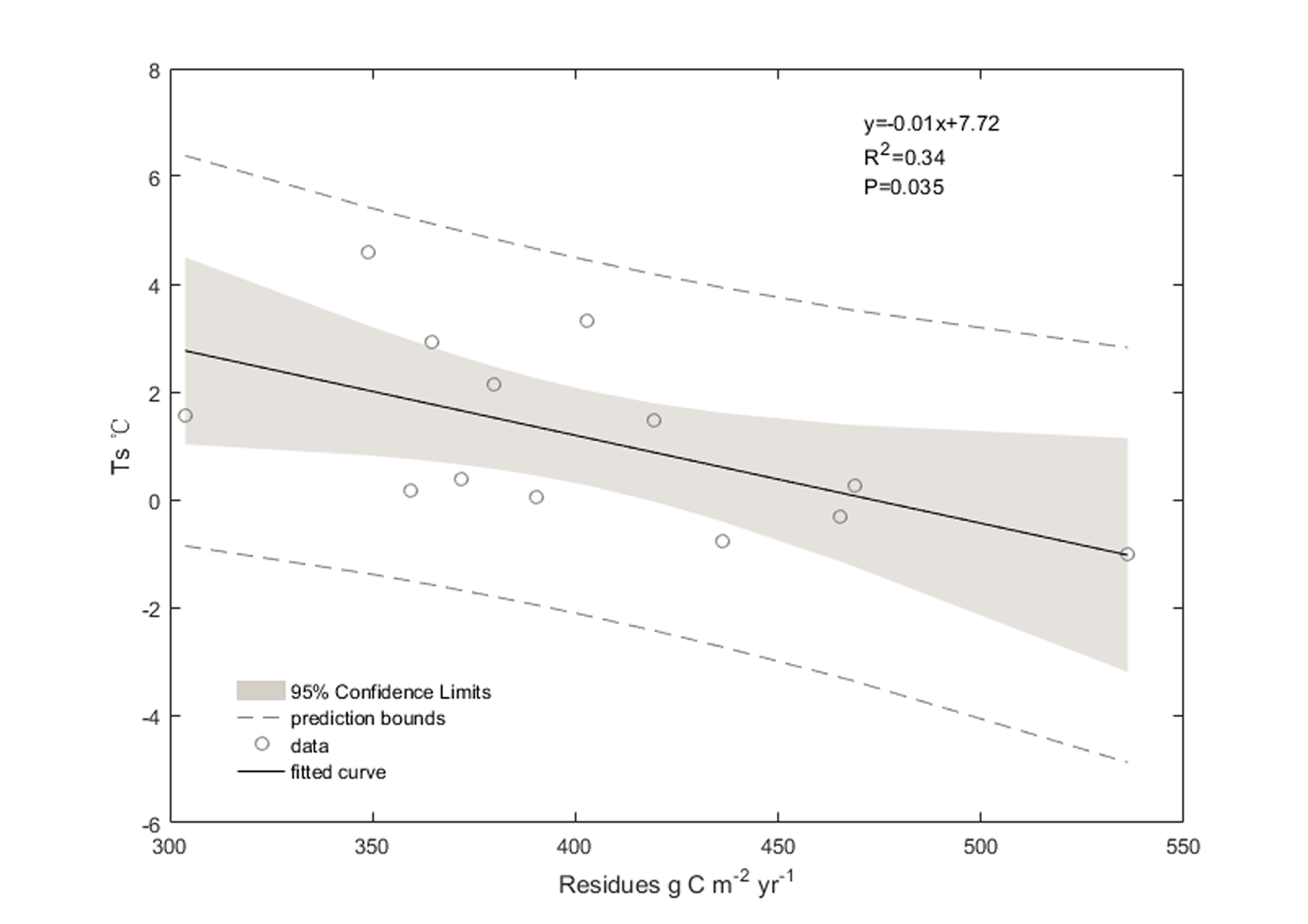

Supplement: S4 Fig — Grey area was 95% confidence limits. Grey dotted lines were prediction bounds. Black line was the fitted curve. (TIF) [file pone.0237684.s010.tif]

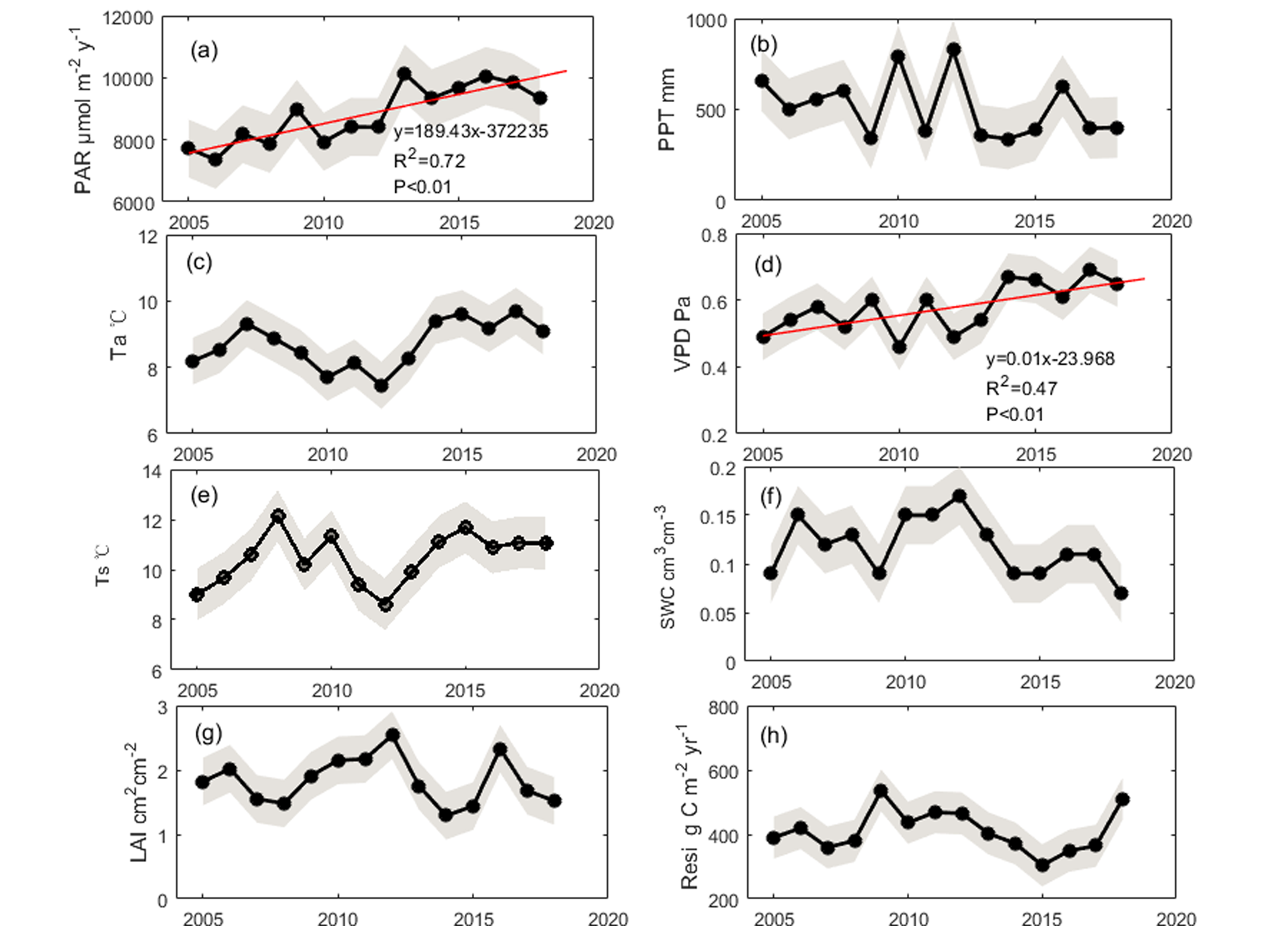

Supplement: S5 Fig — Annual values of PAR(a), PPT(b), Ta(c), VPD(d), Ts(e), SWC(f), LAI(g) and Resi(h). The red line indicate that there was a significant linear regression with time. Grey area was 95% confidence limits. (TIF) [file pone.0237684.s011.tif]

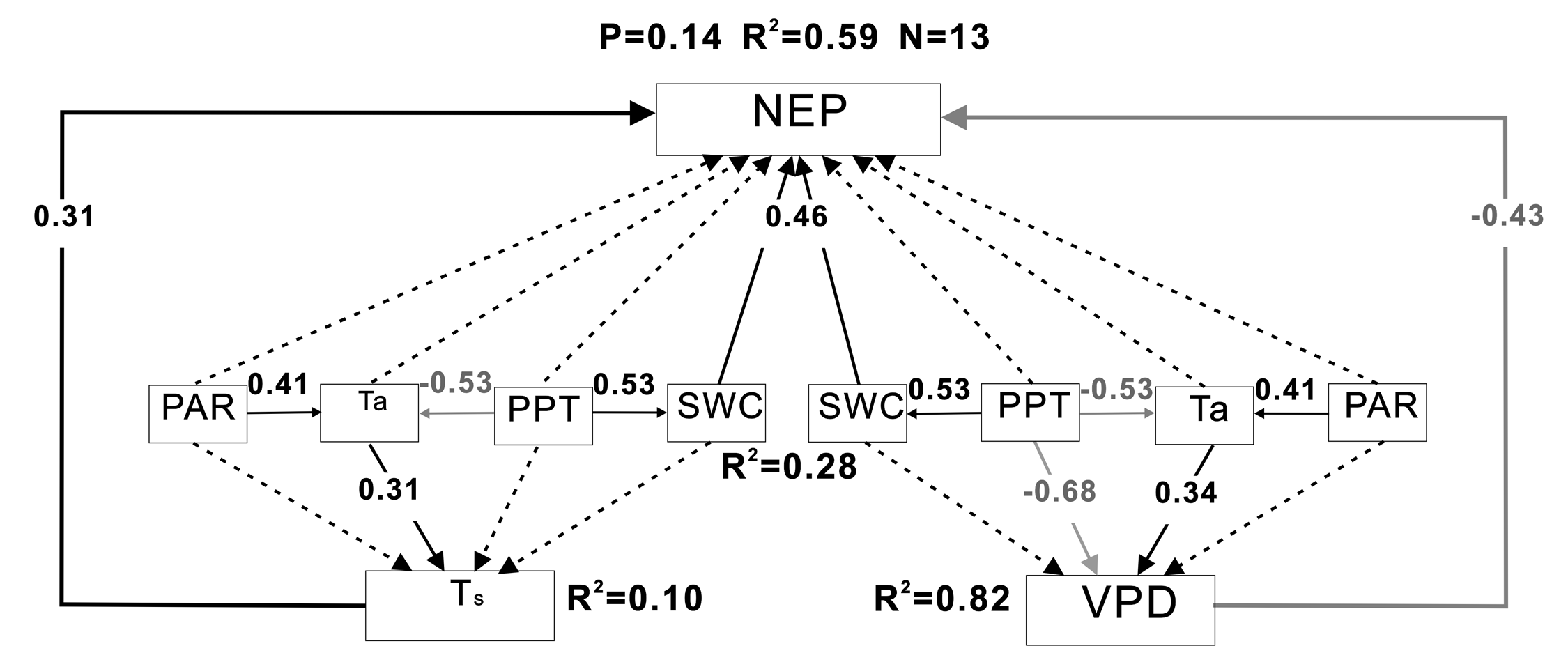

Supplement: S6 Fig — Black arrows indicate significant positive relationships while gray arrows indicate significant negative relationships (P < 0.05). Numbers adjacent to arrows are path coefficients and indicative of the effect size of the relationship. The proportion of variance explained (R2) appears alongside every response variable in the model. (TIF) [file pone.0237684.s012.tif]
